# Neurobiological, Genetic, and Epigenetic Foundations of Eating Disorders in Youth

**DOI:** 10.3390/children11030274

**Published:** 2024-02-23

**Authors:** Luca Cerniglia

**Affiliations:** Faculty of Psychology, International Telematic University Uninettuno, 00186 Rome, Italy; luca.cerniglia@uninettunouniversity.net; Tel.: +39-0669201

Eating disorders (EDs), encompassing conditions such as anorexia nervosa, bulimia nervosa, and binge eating disorder, represent a significant public health concern, particularly among children and adolescents. These disorders are characterized by severe disturbances in eating behaviors and related thoughts and emotions [1,2,3]. The prevalence of EDs in youth is alarming (especially after the COVID-19 pandemic) [4,5,6,7,8], with increasing incidence rates and the early age of onset highlighting the urgency for effective prevention and intervention strategies. Understanding the neurobiological, genetic, and epigenetic underpinnings of these disorders is crucial for developing targeted treatments and improving outcomes for affected individuals.

The neurobiological basis of eating disorders in children and adolescents is a complex interplay of brain structure and function. Recent research has begun to unravel how variations in brain morphology, such as cortical thickness, surface area, and subcortical grey matter volume, are associated with ED symptomatology. For instance, a study within the Adolescent Brain and Cognitive Development cohort found that genetic risk for a high body mass index (BMI) and anorexia nervosa had distinct neural correlates, suggesting that the brain’s structure could underpin specific ED-related psychopathology symptoms [9,10,11,12,13,14,15]. This research indicates that neurobiological factors, including altered cortical thickness in canonical brain networks, may shape disordered eating earlier in life than previously recognized, underscoring the importance of early detection and intervention.

Genetic predisposition plays a significant role in the susceptibility to eating disorders. The development of polygenic scores for anorexia nervosa has shed light on the genetic risk factors associated with this condition. Interestingly, these scores were found to be unrelated to psychopathology factors, suggesting a complex relationship between genetic predisposition and the manifestation of ED symptoms [16]. Furthermore, the differential genetic risk between high BMI and anorexia nervosa, and their distinct neural correlates, highlight the specificity of genetic influences on EDs [17]. Such findings emphasize the need for a nuanced understanding of genetic factors in the context of EDs, which could inform personalized approaches to treatment and prevention.

Epigenetics, the study of how environmental factors influence gene expression without altering the DNA sequence, offers valuable insights into the etiology of eating disorders. Episodes of overeating, dieting, and psychological distress during critical developmental periods, such as puberty and adolescence, can lead to the epigenetic dysregulation of key hormonal axes, impacting the risk of developing EDs. For example, polycystic ovary syndrome, an endocrine condition linked to eating disorders, has been proposed to be induced by such epigenetic alterations [18]. Additionally, the association between co-methylation modules, genetic polymorphisms, and clinical features in adolescents with EDs suggests that genetic variations can alter epigenetics, reflecting variations in clinical features [19]. These findings underscore the intricate relationship between genetic, environmental, and epigenetic factors in the pathogenesis of EDs.

The development of eating disorders in youth is best understood through the lens of the interaction between neurobiological, genetic, and epigenetic factors. This multifaceted approach acknowledges that EDs result from a complex interplay of brain development, genetic predisposition, and environmental influences, all of which contribute to the individual’s risk profile. For instance, the distinct neural correlates associated with genetic risk for high BMI and anorexia nervosa illustrate how specific genetic and neurobiological factors converge to influence ED symptomatology. Such insights are crucial for developing targeted interventions that address the underlying causes of EDs, rather than merely treating their symptoms.

Understanding the neurobiological, genetic, and epigenetic foundations of eating disorders opens new avenues for prevention and treatment. The early detection of individuals at risk, based on their genetic and neurobiological profiles, could enable the implementation of preventative measures before the full-blown development of EDs. Moreover, personalized treatment approaches, tailored to the individual’s specific genetic and neurobiological risk factors, hold promise for more effective interventions. For example, therapies that target the altered brain structures or functions associated with EDs could offer novel treatment pathways.

Despite significant advances in our understanding of the neurobiological, genetic, and epigenetic underpinnings of eating disorders, much remains to be explored. Future research should aim to elucidate the causal relationships between these factors and EDs, employing longitudinal studies that track individuals over time. Additionally, the integration of emerging technologies, such as neuroimaging and genomics, will be crucial for uncovering the complex mechanisms underlying EDs. Such efforts will not only enhance our understanding of these disorders but also pave the way for innovative treatment and prevention strategies.

**Groundbreaking advances in recent years**

In recent years, the field of eating disorders (EDs) has witnessed several breakthrough discoveries, particularly in understanding the neurobiological, genetic, and epigenetic factors contributing to these complex conditions. These advances offer new insights into the pathophysiology of EDs and open avenues for novel therapeutic strategies.

One of the most significant areas of advancement have been in the genetic underpinnings of EDs. Genome-wide association studies (GWASs) have begun to identify specific genetic variants associated with an increased risk of developing EDs. For instance, research has highlighted the role of the serotonin receptor (5-HT2AR) and brain-derived neurotrophic factor (BDNF) genes in susceptibility to EDs. These findings confirm the genetic contribution to EDs and suggest that alterations in neurotransmitter systems and neurotrophic signaling are involved in the etiology of these disorders [20].

Epigenetics, the study of changes in gene expression without alterations in the DNA sequence, has emerged as a crucial area of research in EDs. Studies have shown that environmental factors can lead to epigenetic modifications that influence the risk of developing EDs. For example, malnutrition and stress, common in individuals with anorexia nervosa, can induce reversible epigenetic alterations that affect gene expression related to mental status, metabolism, and immunity [21,22,23,24]. These findings underscore the complex interplay between genetic predisposition and environmental factors in the development of EDs.

Research into the neurobiology of EDs has provided valuable insights into the brain mechanisms underlying these disorders. Advances in neuroimaging techniques have allowed for the exploration of structural and functional brain changes in individuals with EDs. Studies have identified alterations in brain regions involved in reward processing, emotional regulation, and decision making, contributing to the characteristic behaviors observed in EDs [25].

Emerging research has also focused on the gut–brain axis and the role of the microbiome in EDs. Dysbiosis, or imbalances in the gut microbiota, has been linked to the development of EDs. The microbiome may influence brain function and behavior through various pathways, including the immune system, neurotransmitter production, and the vagus nerve. This line of research suggests that targeting the microbiome could offer new therapeutic options for individuals with EDs [26,27].

The use of induced pluripotent stem cells (iPSCs) combined with CRISPR-Cas9 technology represents a groundbreaking approach to studying EDs. This method allows for the generation of specific neuronal cell subtypes from human somatic samples, providing a powerful tool to investigate the cellular and molecular changes underlying EDs [28,29,30,31,32,33,34].

Eating disorders in children and adolescents represent a complex interplay of neurobiological, genetic, and epigenetic factors. Understanding these foundational aspects is crucial for developing targeted prevention and treatment strategies. As research continues to unravel the intricate mechanisms underlying EDs, there is hope for more effective interventions that can improve outcomes for affected individuals. The integration of neurobiological, genetic, and epigenetic insights into clinical practice holds the promise of transforming the landscape of eating disorder treatment, moving towards a more personalized and effective approach.
